# A Novel Visual Sensor Stabilization Platform for Robotic Sharks Based on Improved LADRC and Digital Image Algorithm

**DOI:** 10.3390/s20144060

**Published:** 2020-07-21

**Authors:** Jie Pan, Pengfei Zhang, Jincun Liu, Junzhi Yu

**Affiliations:** 1 State Key Laboratory for Turbulence and Complex Systems, Department of Mechanics and Engineering Science, BIC-ESAT, College of Engineering, Peking University, Beijing 100871, China; panjie@pku.edu.cn (J.P.); liujincun@pku.edu.cn (J.L.); 2 State Key Laboratory of Management and Control for Complex Systems, Institute of Automation, Chinese Academy of Sciences, Beijing 100190, China; zhangpengfei2017@ia.ac.cn; 3 School of Artificial Intelligence, University of Chinese Academy of Sciences, Beijing 100049, China

**Keywords:** visual sensor, stabilization platform, improved LADRC, digital technique, robotic fish

## Abstract

Autonomous underwater missions require the construction of a stable visual sensing system. However, acquiring continuous steady image sequences is a very challenging task for bionic robotic fish due to their tight internal space and the inherent periodic disturbance caused by the tail beating. To solve this problem, this paper proposes a modified stabilization strategy that combines mechanical devices and digital image techniques to enhance the visual sensor stability and resist periodic disturbance. More specifically, an improved window function-based linear active disturbance rejection control (LADRC) was utilized for mechanical stabilization. Furthermore, a rapid algorithm with inertial measurement units (IMUs) was implemented for digital stabilization. The experiments regarding mechanical stabilization, digital stabilization, and target recognition on the experimental platform for simulating fishlike oscillations demonstrated the effectiveness of the proposed methods. The success of these experiments provides valuable insight into the construction of underwater visual sensing systems and also establishes a solid foundation for the visual applications for robotic fish in dynamic aquatic environments.

## 1. Introduction

Bionics have recently attracted great interest in various fields such as robotics, materials science, and architecture. In the area of underwater robotics, the high mobility and stealth of fish have inspired a generation of bionic robotic fish [1], which combines the merits of fish and the functions of autonomous underwater vehicles (AUVs). A variety of robotic fish prototypes have been developed and their maneuverability and practicality have been investigated in recent years [2,3,4,5,6,7].

Visual sensors are essential for robotic fish so that they can accomplish complex tasks like detecting obstacles and avoiding them [8]; this generally involves a trade-off between a feasible technology and the bionic prototype. Compared with ultrasound sensors, visual devices are easier to integrate into miniaturized robotic fish, and nowadays, robotic fish based on visual sensors have been applied to a variety of missions. For instance, Yu et al., designed an embedded monocular visual sensor to modulate control parameters, which facilitated environmental perception and goal-directed-based swimming [9]. Stefanini et al., developed a binocular visual sensor mounted on a robotic lamprey, which provided a wide field of images and sent back the motion parameters [10]. Ricardo et al., proposed a novel vision system for navigation under poor visibility conditions in a semi-structured environment [11]. Zheng et al., described an autonomous vision-based robotic fish for underwater competition [12]. Although a few visual systems have been designed for robotic fish, the application of visual sensors is relatively new and simplistic because of the poor image quality. Compared with land images, acquiring stable and high-quality underwater images with robotic fish is more challenging. Besides the adverse underwater lighting environment, the most serious problem is that the camera shake caused by the motion of the robotic fish causes image motion blur and an unstable field of view. This has resulted in great challenges for the visual-based applications of the robotic fish. Therefore, the construction of a visual sensor stabilization platform is core to the future development of this technology.

Other types of machine systems such as unmanned aerial vehicles (UAVs) and quadruped robots have made great progress compared with the stabilization of robotic fish visual sensors. For example, Lim et al., designed a digital stabilization algorithm of higher-level visual tasks for UAV [13]. High-speed operation was the key objective, and this method made use of switching between rigid and similarity transformation, optical flow-based motion estimation, and multi-threading. Kim and Choi used an extended neural network method to detect object azimuth for UAV surveillance [14]. They used a specially equipped GoPro, which worked well, however, it needed a large interspace. Bazeille et al., constructed a visual system that mainly relied on a rigid pan and tilt unit (PTU) to achieve visual stabilization for quadruped robots [15].

**Figure 1:**
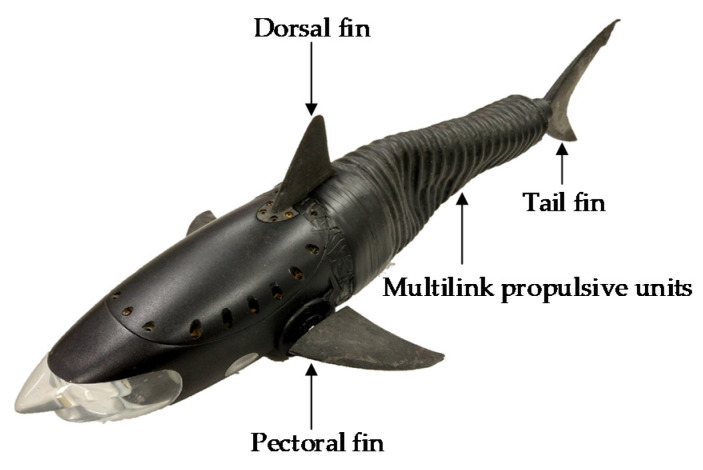
The shark-like robotic fish prototype [16].

However, the abovementioned methods do not seem to be feasible for a self-propelled robotic fish due to the limited space, computational time, and other factors. The disturbance of the robotic fish visual sensor is caused mainly by the periodic oscillation of the fish tail during straight swimming, as is shown in Figure 1. The tail oscillation not only induces a recoil motion in the yaw direction but also a sway in the roll axis owing to the non-uniform mass distribution. Thus, the visual sensor fixed on the robotic fish is also affected by this oscillation. Many studies have been conducted to address this issue. For example, because the complex equipment is difficult to mount on the previously developed shark-like robotic fish, a visual sensor stabilizer with a cascade control approach was developed [16,17]. However, its stabilization effect was limited because it was a one degree-of-freedom (DOF) visual sensor stabilization system that was only designed to stabilize the yaw direction. On the basis of their predecessors, Zhang et al., designed a compact 2-DOF stabilization gimbal for the visual sensor using the active disturbance rejection control (ADRC) algorithm [18]. A crucial drawback is that it is twice the size of the 1-DOF stabilization system, which makes it unsuitable in small robotic fish. Lou et al., proposed a new kinematic model of a bionic robotic fish to restrain the head swinging [19]. Unfortunately, it was unable to stabilize the visual sensors.

In order to strike a balance between the volume of the device and stabilization performance, this paper proposes a 1-DOF stabilization platform for robotic shark visual sensors that combines mechanical stabilization and digital image technology. First, the visual sensor stabilization was simplified as a 2-D stabilization problem ignoring the pitch channel, and the expression of the controlled quantity was derived based on the kinematic relationship. Considering the tight space, mechanical and digital stabilization processes were adopted for the yaw and roll channels, respectively. To verify the effectiveness of the proposed system, an experimental platform was developed to mimic fishlike propulsion and maneuvering. The mechanical stabilization, digital stabilization, and target recognition results revealed that the stabilization system, with an improved linear active disturbance rejection control (LADRC) algorithm and a rapid digital stabilization algorithm using inertial measurement units (IMUs) can significantly enhance the visual sensor stabilization effects. The main contributions of this paper are summarized as follows:

- An improved window function-based LADRC algorithm was proposed for mechanical stabilization, which performs better than the general LADRC algorithm in 3-D motion.
- The IMU measurement data was applied as an estimation of motion to reduce the dependence of digital stabilization on image features, which guarantees real-time performance and is more suitable for underwater application.
- A stabilization scheme combining mechanical stabilization and digital stabilization employed for small robotic fish visual sensors was proposed. Compared to existing systems, the platform possesses a more compact structure and a better stabilization effect. Experiments verified that it can significantly improve the accuracy of target detection tasks. Remarkably, it offers valuable insight into the construction of underwater visual sensing platforms and lays the foundation for an underwater visual application such as target tracking of untethered robotic fishes with a stabilization system.

The remainder of this paper is structured as follows. The problem formulation for the visual sensor stabilization platform is described in Section 2. The control system is detailed in Section 3, including the LADRC, the switching system, and the improved LADRC. The overall 1-DOF stabilization platform framework and the stability of the proposed improved LADRC applied in the platform are discussed in Section 4. The mechanical stabilization, digital stabilization, and target recognition experiments are presented in Section 5. Finally, in Section 6, we present the conclusions and discuss future work.

## 2. Problem Formulation

Constructing a stable visual sensing system is undoubtedly the key factor for several underwater tasks such as target recognition and tracking. However, it is not easy to achieve when the robotic fish is swimming. Typically, three traditional image stabilization solutions have been used: mechanical stabilization, digital stabilization, and optical stabilization [20]. Of the traditional solutions, mechanical stabilization is prioritized because of its outstanding stability. Thus, due to the small space in the interior of the shark-like robotic fish head, a 1-DOF visual sensor stabilization system was employed to solve the issue of periodic disturbance.

**Figure 2:**
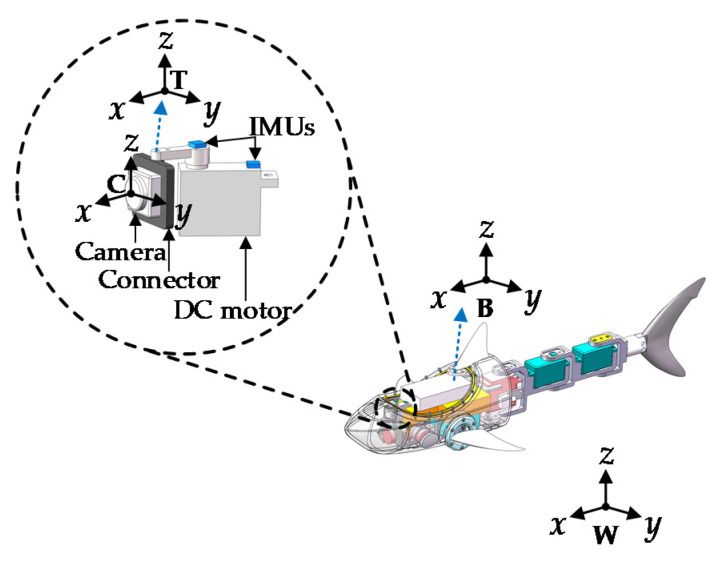
Schematic diagram of the one degree-of-freedom (1-DOF) visual sensor stabilization platform in the shark-like robotic fish.

The shark-like robotic fish was developed in our previous work [16]. It is 483 mm long, 208 mm wide, and 125 mm high. For the sake of clarity, the components of the 1-DOF visual sensor stabilization platform is illustrated in Figure 2, and the visual sensor used in this study is the monocular camera. Specifically, the four coordinate systems are defined, whereby frames *T* and *C* fixed on the camera are related to the desired camera attitude and the current camera attitude, respectively. The body frame *B* is fastened at the center of gravity of the fish and corresponds to the attitude of the shark-like robotic fish. The world frame *W* is consistent with the initial body attitude. Then, the problem can be simplified as

(1) $$R_{C}^{T}=R_{C}^{W}\cdot R_{W}^{T}\to I$$

where $R_{A}^{B}$ denotes the rotation matrix from frame *A* to *B*, and *I* is an identity matrix. Notably, $R_{W}^{T}$ and $R_{C}^{T}$ act as input and output variables, respectively. Thus, the problem becomes how to achieve the approach of $R_{C}^{T}$ to *I* by adjusting the intermediate variable $R_{C}^{W}$.

As the robotic fish is capable of 3D swimming, only the roll and yaw angles need to be compensated for. The reason is that the pitch angle does not need to be counteracted in diving and ascending, and sometimes it can be omitted. Therefore, the 1-DOF visual sensor stabilization system is designed to compensate for the roll and yaw disturbances, and $R_{C}^{T}$ can be expressed as

(2) $$\begin{array}{ll} R_{C}^{T} & =Rot\left(Z_{C},\psi_{CT}\right)Rot\left(X_{C},\theta_{CT}\right)\\ & =Rot\left(Z_{C},\psi_{C}\right)Rot\left(X_{C},\theta_{C}\right)Rot{\left(X_{T},\theta_{T}\right)}^{T}Rot{\left(Z_{T},\psi_{T}\right)}^{T} \end{array}$$

where $\psi_{CT}$ and $\theta_{CT}$ represent the yaw and roll error angles, respectively. $\psi_{C}$ and $\theta_{C}$ denote the current yaw and roll angles. $\psi_{T}$ and $\theta_{T}$ represent the target yaw and roll angles. The problem is equivalent to:

(3) $$Rot\left(Z_{C},\psi_{C}\right)Rot\left(X_{C},\theta_{C}\right)=Rot\left(Z_{C},\psi_{CT}\right)Rot\left(X_{C},\theta_{CT}\right)Rot\left(Z_{T},\psi_{T}\right)Rot\left(X_{T},\theta_{T}\right)$$

where Rot(Z,ψ) and Rot(X,θ) are construed as

(4) $$Rot\left(Z,\psi \right)=\left[\begin{array}{ccc} \cos\psi & -\sin\psi & 0\\ \sin\psi & \cos\psi & 0\\ 0 & 0 & 1 \end{array}\right],$$

(5) $$Rot\left(X,\theta \right)=\left[\begin{array}{ccc} 1 & 0 & 0\\ 0 & \cos\theta & -\sin\theta \\ 0 & \sin\theta & \cos\theta \end{array}\right].$$

Ultimately, $\psi_{C}$ and $\theta_{C}$ can be determined by $\psi_{CT}$, $\theta_{CT}$, $\psi_{T}$, and $\theta_{T}$.

In practice, the mechanical stabilization is operated in the yaw direction. However, since there is not enough space to support the mechanical stabilization device in the rolling direction, the roll angle needs to be solved by adding other technologies. Thus, a rapid digital stabilization algorithm combined with IMUs was proposed as described in in Section 4.

## 3. Control System Design

The control system was developed to maintain the visual sensor platform stability in the yaw direction during the motion, with a particular focus on the control error and dynamic performance. In particular, the use of ADRC to compensate for the disturbance has a better effect than traditional PD control. Therefore, considering the nonlinearity and parameter tuning issues, LADRC and the switching system combined LADRC with nonlinear ADRC (NLADRC) and this is discussed in this section [21]. In addition, it is worth noting that the total disturbance estimated by extended state observer (ESO) is derived from the internal and external disturbances, which can have detrimental and beneficial effects, respectively [22,23]. Therefore, a disturbance characterization indicator (DCI) was designed to determine whether to use or compensate for the total disturbance. The improved LADRC will also be discussed in this section.

### 3.1. LADRC

Although nonlinear ADRC (NLADRC) is excellent for nonlinear problems, it is difficult to tune the parameters [21,24,25]. Consequently, LADRC consisting of a tracking differentiator (TD), a linear ESO (LESO), and a linear state error feedback (LSEF) law was employed [26].

In this situation, the input angle and angular velocity are retrieved by IMUs, in lieu of the TD segment. Thus, the corresponding algorithm is described by

(6) $$\left\{ \begin{array}{l} {\tilde{e}}_{1}\left(t\right)=z_{1}\left(t\right)-y\left(t\right)\\ {\dot{z}}_{1}\left(t\right)=z_{2}\left(t\right)-\beta_{01}\varphi_{1}\left({\tilde{e}}_{1}\right)\\ {\dot{z}}_{2}\left(t\right)=z_{3}\left(t\right)-\beta_{02}\varphi_{2}\left({\tilde{e}}_{1}\right)+bu\left(t\right)\\ {\dot{z}}_{3}\left(t\right)=-\beta_{03}\varphi_{3}\left({\tilde{e}}_{1}\right)\\ e_{1}\left(t\right)=v_{1}\left(t\right)-z_{1}\left(t\right)\\ e_{2}\left(t\right)=v_{2}\left(t\right)-z_{2}\left(t\right)\\ u\left(t\right)=u_{0}-z_{3}\left(t\right)/b \end{array}\right.,$$

(7) $$u_{0}=k_{p}e_{1}\left(t\right)+k_{d}e_{2}\left(t\right),$$

(8) $$\varphi_{1}\left({\tilde{e}}_{1}\right)=\varphi_{2}\left({\tilde{e}}_{1}\right)=\varphi_{3}\left({\tilde{e}}_{1}\right)={\tilde{e}}_{1}\left(t\right),$$

(9) $$\beta_{01}=3w_{0},\;\beta_{02}=3w_{0}^{2},\;\beta_{03}=w_{0}^{3}$$

where the description of the formulaic terms is listed below in Table 1.

### 3.2. Switching System

A switching control method for LADRC and NLADRC was proposed by Li et al. [25]. They simplified parameter tuning and took advantage of LADRC with NLADRC. With regard to NLADRC, the corresponding algorithm is shown as

(10) $$\left\{ \begin{array}{l} u_{0}=k_{p}fal\left(e_{1},\alpha_{1},\delta \right)+k_{d}fal\left(e_{2},\alpha_{2},\delta \right)\\ \varphi_{1}\left({\tilde{e}}_{1}\right)=fal\left({\tilde{e}}_{1},\alpha_{3},\delta \right)\\ \varphi_{2}\left({\tilde{e}}_{1}\right)=fal\left({\tilde{e}}_{1},\alpha_{4},\delta \right)\\ \varphi_{3}\left({\tilde{e}}_{1}\right)=fal\left({\tilde{e}}_{1},\alpha_{5},\delta \right) \end{array}\right.,$$

(11) $$\beta_{01}=3w_{0},\;\beta_{02}=3w_{0}^{2},\;\beta_{03}=w_{0}^{3},$$

(12) $$\varphi_{i}\left({\tilde{e}}_{1}\right)=fal\left({\tilde{e}}_{1},\alpha_{i},\delta \right)=\left\{ \begin{array}{r} \frac{{\tilde{e}}_{1}}{\delta^{1-\alpha_{i}}},\left|{\tilde{e}}_{1}\right|\leq \delta \\ {\left|{\tilde{e}}_{1}\right|}^{\alpha_{i}}sgn\left({\tilde{e}}_{1}\right),\left|{\tilde{e}}_{1}\right|>\delta \end{array}\right.$$

which includes a nonlinear ESO (NLESO) and a nonlinear state error feedback (NLSEF) law from Equation (6).

The switching conditions include t>T, $z_{3}\left(t\right)>M$, and $\left|{\tilde{e}}_{1}\right|>1$. As long as one of the three conditions is satisfied, the system switches from NLADRC to LADRC, and vice versa.

### 3.3. Improved LADRC

The disturbance is divided into two types, one has a positive impact, namely, beneficial disturbance, the other has a negative impact, namely, detrimental disturbance. Because the disturbance has a dual character, the visual sensor platform is not required to reject all of the disturbance. Afterwards, the total disturbance is selectively utilized or compensated for by the motor to improve stability. Sun et al., reported the vigorous function of the disturbance in control engineering practice for the first time [22], and a DCI was proposed that is described as

(13) $$\left\{ \begin{array}{c} {\hat{e}}_{1}\left(t\right)=y\left(t\right)-v_{1}\left(t\right)\\ J=sgn\left({\hat{e}}_{1}\right)\cdot sgn\left(z_{3}\right) \end{array}\right.$$

which is essentially a judgment statement. It is obvious that J<0 means that the total disturbance $z_{3}\left(t\right)$ observed by LESO has the opposite sign with angle error ${\hat{e}}_{1}\left(t\right)$ and vice versa. In reality, ${\hat{e}}_{1}\left(t\right)<0$ indicates that the current angle is smaller than the target angle. Thus, the disturbance is beneficial if $z_{3}\left(t\right)>0$ during the stabilization process. On the contrary, the disturbance is harmful if $z_{3}\left(t\right)<0$. Similarly, another situation where ${\hat{e}}_{1}\left(t\right)>0$ can be analyzed. On the whole, the disturbance is beneficial when J>0, and vice versa. As stated previously, the linear control law is adjusted by

(14) $$u\left(t\right)=k_{p}e_{1}\left(t\right)+k_{d}e_{2}\left(t\right)-Jz_{3}\left(t\right)/b.$$

**Figure 3:**
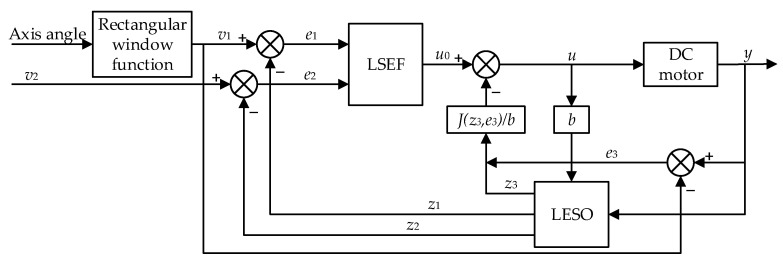
Stabilization block diagram of the improved linear active disturbance rejection control (LADRC) control, where *θ* and *y* are measured by inertial measurement units (IMUs), respectively. One of them is fixed on the camera and the other is fixed on the head of the shark-like robotic fish.

With respect to the ratio of disturbance compensation and utilization, the choice is 100%. A continuous function was designed to avoid the chattering caused by the sign function, which is given by:

(15) $$sgn\left(x\right)=\frac{x}{\sqrt{x^{2}+\varepsilon^{2}}}$$

where ε is a small positive number. Based on the merits of DCI and ESO, an improved LADRC algorithm combining Equations (6), (9), (13), (14), and (15) was proposed, in conjunction with the block diagram indicated in Figure 3. This is particularly effective when the robotic fish is turning because it fully utilizes the influence of disturbance.

### 4. 1-DOF Visual Sensor Stabilization System Design

In order to achieve the approach of $R_{C}^{T}$ to *I*, mechanical stabilization is utilized to compensate for disturbances in the yaw direction as the $Rot\left(Z_{C},\psi_{C}\right)$, and digital stabilization is used to compensate for disturbances in the roll direction as the $Rot\left(X_{C},\theta_{C}\right)$. In addition, $Rot\left(Z_{T},\psi_{T}\right)$ and $Rot\left(X_{T},\theta_{T}\right)$ act as input terms, while $Rot\left(Z_{C},\psi_{CT}\right)$ and $Rot\left(X_{C},\theta_{CT}\right)$ play a role in the feedback.

**Figure 4:**
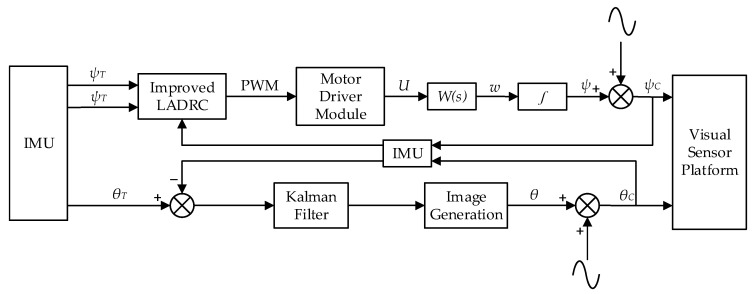
Schematic diagram of the 1-DOF visual sensor stabilization platform in the shark-like robotic fish.

The visual system framework of a 1-DOF visual sensor stabilization platform is depicted in Figure 4. Specifically, it is updated with an improved LADRC utilizing the total disturbance estimated by linear extended state observer (LESO) to compensate in the yaw direction, and coupled with a rapid digital stabilization algorithm to stabilize the roll direction. Considering the limited head space of the robotic shark, the angle of the vision sensor is collected by IMU instead of a laser diode [26].

### 4.1. Mechanical Stabilization

A servomotor with the control board removed was utilized as a controlled object for a fast response, and its transfer function is formulated as

(16) $$\frac{W\left(s\right)}{U\left(s\right)}=\frac{K_{m}}{\left(T_{e}s+1\right)\left(T_{m}s+1\right)}$$

where $K_{m}$ is the motor gain constant, $T_{e}$ is the field time constant, and $T_{m}$ is the mechanical time constant. Owing to $T_{m}>10T_{e}$, the field time constant is usually neglected. Thus, the dynamic model of the controlled object without uncertainties and disturbances is written in the state space in the following form:

(17) $$\left\{ \begin{array}{l} \dot{x}\left(t\right)=Ax\left(t\right)+Bu\left(t\right)+C\\ y\left(t\right)=Dx\left(t\right) \end{array}\right.$$

where $A=\left[\begin{array}{cc} 0 & 1\\ 0 & -\frac{1}{T_{m}} \end{array}\right]$, $B=\left[\begin{array}{c} 0\\ \frac{K_{m}}{T_{m}} \end{array}\right]$, $C=\left[\begin{array}{c} 0\\ -\frac{K_{e}}{T_{m}}M_{e} \end{array}\right]$, $D=\left[\begin{array}{cc} 1 & 0 \end{array}\right]$, $x={\left[\begin{array}{cc} x_{1}\left(t\right) & x_{2}\left(t\right) \end{array}\right]}^{T}$,$K_{e}$ is the load coefficient, and $M_{e}$ is the external load moment. Combined with u(t) calculated by the improved LADRC, the mechanical stabilization in the yaw direction is achieved.

### 4.2. Stability Analysis

The proposed improved LADRC for the controlled DC motor is bounded-input bounded-output (BIBO) stability. The analysis is divided into two parts, one is the stability of the LESO, and the other is the stability of the closed-loop system.

**Theorem** **1.** *Suppose a general error dynamic system*

(18) $$\dot{\eta }=M\eta +G\left(\eta \right)$$

*where M is a Hurwitz matrix and G(η) is bounded,* $\eta \in {\mathbb{R}}^{n}$ *, and* $M\in {\mathbb{R}}^{n\times n}$ *, the error* η *is uniformly ultimately bounded [27,28,29,30].*

#### 4.2.1. Convergence of LESO

Firstly, the augmented state space of (17) with the expanded state $x_{3}$ can be reformulated by

(19) $$\left\{ \begin{array}{l} {\dot{x}}_{1}\left(t\right)=x_{2}\left(t\right)\\ {\dot{x}}_{2}\left(t\right)=x_{3}\left(t\right)+bu\\ {\dot{x}}_{3}\left(t\right)=\dot{f} \end{array}\right.$$

where $f=-\frac{1}{T_{m}}x_{2}\left(t\right)-\frac{K_{e}}{T_{m}}M_{e}+\left(\frac{K_{m}}{T_{m}}-b\right)u$ serves as the total disturbance.

Let ${\tilde{e}}_{i}=z_{i}-x_{i}$, i=1,2,3. Thus, the error dynamics in the observer can be shown as

(20) $$\dot{\tilde{e}}=\tilde{A}\tilde{e}+\tilde{B}\dot{f}$$

where $\tilde{A}=\left[\begin{array}{ccc} -3w_{0} & 1 & 0\\ -3w_{0}^{2} & 0 & 1\\ -w_{0}^{3} & 0 & 0 \end{array}\right]$, $\tilde{B}=\left[\begin{array}{l} \begin{array}{c} 0\\ 0 \end{array}\\ -1 \end{array}\right]$, $\tilde{e}={\left[\begin{array}{ccc} {\tilde{e}}_{1} & {\tilde{e}}_{2} & {\tilde{e}}_{3} \end{array}\right]}^{T}$.

**Assumption** **1.** *The differentiation of the augmented state f is bounded $\left|\dot{f}\right|\leq B_{1}$, where $B_{1}$ is a positive constant.*

The eigenvalues of $\tilde{A}$ is $-w_{0}$, that is, the real part is negative. Thus, the matrix $\tilde{A}$ is Hurwitz. Combined with the reasonable Assumption 1, the observed estimation error $\tilde{e}$ is uniformly ultimately bounded, and its upper bound decreases with the observer bandwidth $w_{0}$ [31].

#### 4.2.2. Stability of the Closed-Loop System

Let ${\hat{e}}_{i}=x_{i}-v_{i}$, i=1,2. Specifically,

(21) $$\begin{array}{ll} {\dot{\hat{e}}}_{2} & ={\dot{x}}_{2}-{\dot{v}}_{2}\\ & =f+bu-{\dot{v}}_{2} \end{array}$$

Using the control input (14),

(22) $${\dot{\hat{e}}}_{2}=f+k_{p}be_{1}+k_{d}be_{2}-Jz_{3}-{\dot{v}}_{2}$$

Then, $e_{i}=v_{i}-z_{i}=-{\hat{e}}_{i}-{\tilde{e}}_{i}$ and it is considered that

(23) $${\dot{\hat{e}}}_{2}=-k_{p}b{\hat{e}}_{1}-k_{d}b{\hat{e}}_{2}-k_{p}b{\tilde{e}}_{1}-k_{d}b{\tilde{e}}_{2}-Jz_{3}+f-{\dot{v}}_{2}$$

Thus, the error dynamics in the closed-loop system can be expressed as

(24) $$\dot{\hat{e}}=\hat{A}\hat{e}+\hat{B}h$$

where $\hat{A}=\left[\begin{array}{cc} 0 & 1\\ -bk_{p} & -bk_{d} \end{array}\right]$, $\hat{B}=\left[\begin{array}{ccccc} 0 & 0 & 0 & 0 & 0\\ -bk_{p} & -bk_{d} & -J & 1 & -1 \end{array}\right]$, $h={\left[\begin{array}{ccccc} {\tilde{e}}_{1} & {\tilde{e}}_{2} & z_{3} & f & {\dot{v}}_{2} \end{array}\right]}^{T}$, $\hat{e}={\left[\begin{array}{cc} {\hat{e}}_{1} & {\hat{e}}_{2} \end{array}\right]}^{T}$, J∈(-1,1).

**Assumption** **2.** *The target acceleration ${\dot{v}}_{2}$ is bounded $\left|{\dot{v}}_{2}\right|\leq B_{2}$, where $B_{2}$ is a positive constant. In addition, the augmented state f is bounded $\left|f\right|\leq B_{3}$, where $B_{3}$ is a positive constant.*

The $k_{p}>0$ and $k_{d}>0$ are satisfied, that is, the matrix $\hat{A}$ is Hurwitz. In addition, due to the BIBO stability of the LESO and the reasonable Assumption 2, the closed-loop system tracking error is uniformly ultimately bounded.

### 4.3. Digital Stabilization

Due to the limited space, digital stabilization is exploited to compensate for insufficient mechanical stabilization. Traditionally, digital stabilization consists of motion estimation, motion compensation, and image generation. Due to the integration of many new technologies, there has been rapid development in this area, including motion estimation, which is the most notable. For instance, Kim et al. (2008) proposed a probability model for global motion estimation to improve computational efficiency [32]. Dong et al. (2019) designed a software algorithm concentrating on motion estimation to obtain an optimal transformation matrix using three steps [33]. After utilizing moment-based speeded-up robust features (MSURF) to obtain original transformation matrix, k-means clustering was used to exclude outliers, and sequential quadratic programming (SQP) was employed to acquire robust results. Li et al. (2020) suggested an optimization method for shaking hand-held adaptive video stabilization [34]. The novelty of this method included decomposing the complicated signal by empirical mode decomposition (EMD), and avoiding over-cutting by over-smoothing with feature-centric EMD. However, due to time delays as well as poor-quality feature points, these methods are not applicable to aquatic environments. Therefore, we decided that the data collected by IMUs should replace motion estimation. Ertürk integrated a Kalman filter into a real time stabilization system to eliminate unintentional motion and retain intentional motion [35]. Specifically, the Kalman filter is a standard real-time filter for motion compensation [36]. Thus, the Kalman filter can be employed to process the angle information and in the end, a stable image sequence is acquired through image generation. Through these steps, digital stabilization can be realized in the roll direction.

## 5. Experimental Results

**Figure 5:**
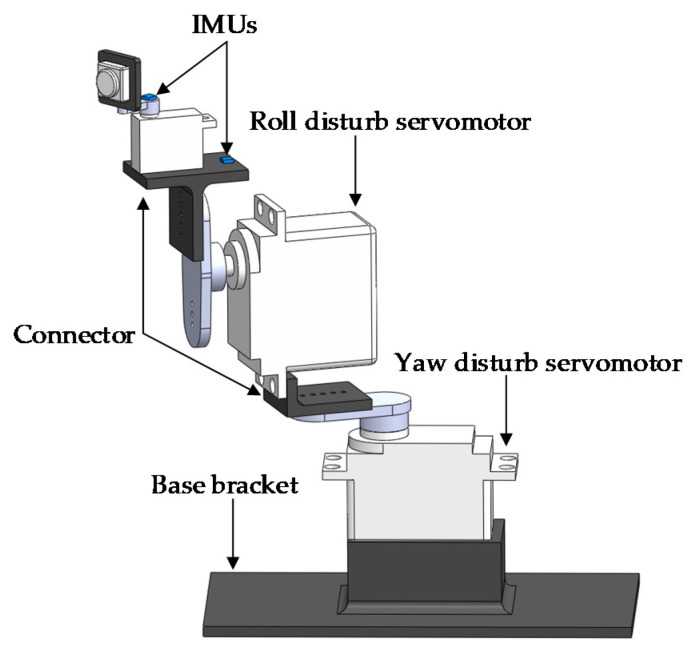
The schematic diagram of the experimental platform.

To verify the effectiveness of the visual sensor stabilization platform, we used an experimental platform that simulates shark-like robotic fish oscillations, as shown in Figure 5. Two servomotors were employed to create the yaw and roll motion, respectively. They all rotated as a sine wave, with the same frequency and different amplitudes. Two IMUs were mounted to play the roles of $R_{C}^{W}$ and $R_{T}^{W}$. One was used to measure the visual system angle, which was attached to the camera. The other was utilized to estimate the central axis position of the shark-like robotic fish, which was fixed to the roll servomotor. This platform mimicked the swimming of the shark-like robotic fish since the motion of the pitch maneuver could be neglected.

### 5.1. Mechanical Stabilization Experiment

The experimental process involved a move linearly, turn right, resume a linear motion, and then this was repeated. One periodic motion was 8 seconds and the turning motion lasted for 2 s. The turning process was controlled by constantly adjusting the central axis of the yaw servomotor. The yaw angle was provided by an IMU fastened onto the roll servomotor. Due to the sine wave around the central axis, the angle cannot be used as the position of the turning central axis. Thus, a rectangular window method played an important role, and took advantage of the sine wave. The selection of the window function length was consistent with a sinusoidal motion period, and the window kept adding the latest angle and eliminated the oldest angle to make an average.

**Figure 6:**
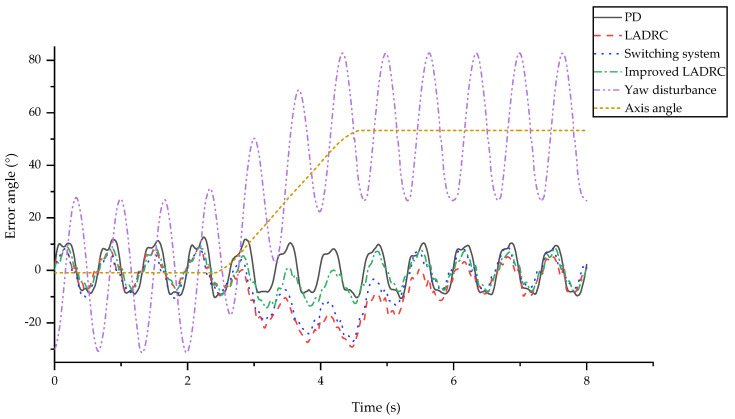
Experimental results of proportion differential (PD) control, LADRC, switching system, improved LADRC, yaw disturbance, and axis angle under the yaw sine wave disturbance with frequency of 1.5 Hz and amplitude of 30°, the roll sine wave disturbance with a frequency of 1.5 Hz an amplitude of 10°, and a turning speed of 25°/s.

Within the context of the visual sensor stabilization platform, close attention was paid to the dynamic performance including the response time. Thus, the traditional proportion differential (PD) algorithm served as a benchmark. It was defined as

(25) $$C\left(s\right)=k_{p}+k_{d}s$$

where *k_p_* and *k_d_* are the proportional and differential coefficient, respectively. LADRC, the switching system, and improved LADRC were also verified to choose the best algorithm. Since the mechanical stabilization targeted the yaw direction, the experimental results were concerned with the yaw angle. The experiments were progressed through a frequency range of 0.5 Hz to 1.5 Hz and we found that the higher the frequency, the greater the differences. Thus, 1.5 Hz was chosen as an example for detailed analysis, and the results are depicted in Figure 6.

The experimental conditions and data processing were as follows. Firstly, the yaw sinusoid amplitude was 30° and the roll sinusoid amplitude was 10°. The difference between them was determined by the actual shark-like robotic fish motion. The turning speed was 25°/s. Then, for the sake of the transparency of the graph, the yaw angle curve was the difference between the camera real-time angle and the central axis angle. Consequently, the curve varied around zero, which means that the zero line was the target position. The axis position change and the sinusoid yaw angle disturbance are also shown in Figure 6, as calculated by the rectangular window function. These intuitive profiles suggest that the most suitable algorithm is the improved LADRC using the nature of the disturbance. In addition, other objective and valuable evaluating indicators are determined by:

(26) $$APR=\frac{1}{N}\sum_{p=1}^{N}\left(y_{pmax}-y_{pmin}\right)$$

(27) $$Range=y_{max}-y_{min}$$

(28) $$MAE=\frac{1}{n}\sum_{i=1}^{n}\left|y_{i}-y_{axis}\right|$$

(29) $$RMSE=\sqrt{\frac{1}{n}\sum_{i=1}^{n}{\left(y_{i}-y_{axis}\right)}^{2}}$$

where *y_pmax_* and *y_pmin_* are the maximum and minimum angles over a period, *y_max_* and *y_min_* are the maximum and minimum angles of the whole process, *y_i_* is the yaw angle of the camera, *y_axis_* is the angle of the target axis, *n* is the number of the sampling points, and *N* is the number of the periods.

The specific evaluation values are listed in Table 2. APR is the average of the range of each period, which illustrates the visual sensor platform stabilization effect and precision. MAE and RMSE are used to judge the deviation of the actual from the ideal yaw angle. The difference between them is that RMSE is more sensitive to unsatisfactory angles.

Based on the obtained experimental results, the following conclusions were drawn:

- The PD method does not show any drift phenomenon, which demonstrates its outstanding dynamic performance. The other algorithms have a significant drift when turning, which indirectly minimizes the range. Although the tracking performance is improved with less drift phenomenon, the excessive APR reduces this advantage.
- All of the indicators for LADRC are almost the biggest compared to the other methods. This is mainly due to the slow response to the axis changes, which is clearly shown in Figure 6. The RMSE is 9.5887°, which indicates that there are many obvious abnormal points. However, it is worth noting that it has the smallest APR, which shows its better control error performance. This phenomenon suggests that the LADRC is the best model when there is only linear motion.
- Although the switching system is slightly improved compared with LADRC, there is a significant gap compared to the improved LADRC, and parameter tuning is more difficult. The stability of the switching system is closely related to tuning of the parameters, which was previously discussed in [25].
- The range, MAE and RMSE of the improved LADRC are much enhanced, compared to the traditional LADRC. Moreover, the APR is almost the same. Thus, the effectiveness of the rational use of disturbance is verified. In particular, the drift phenomenon is not apparent in this method, thus excessive changes in range are avoided.

The profiles demonstrate that the improved LADRC is the best choice, especially because of its particular emphasis on the control error and dynamic performance, which combines the advantages of PD and LADRC. This experiment shows the superiority of the improved LADRC algorithm.

### 5.2. Digital Stabilization Experiment

The purpose of this part was to reduce the distortion of the images caused by the rolling of the fish body, and make the image sequence more conducive to target detection, tracking, and recognition. The method of motion estimation was used to calculate the optimal motion vector based on two adjacent frames from underwater videos [37]. The poor results that were obtained were due to the few valid feature points, and indicated that this method was not useful for the roll maneuver. Additionally, the complex algorithms were not applicable because of their requirements for real time. Consequently, the motion estimation step was changed in order to focus on the roll angle of the IMU, which was fixed on the camera [38]. The accuracy of this alternative approach should be higher. Remarkably, the frequency of the IMUs was much higher than the frame rate of the camera. Thus, multi-threading was utilized to ensure real-time performance. Eventually, the final stable image sequence was obtained with the following Kalman filter and motion compensation. The vision sensor needs to be calibrated before the experiment.

**Figure 7:**
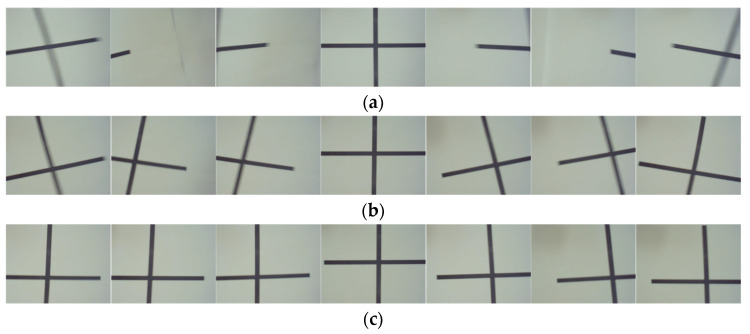
Comparison of the different visual sensor stabilization platform experiments. (**a**) The system without mechanical stabilization and digital stabilization; (**b**) The pure 1-DOF mechanical stabilization system; and (**c**) The system combining mechanical stabilization with digital stabilization.

One cycle image sequence was selected to compare the results for the three cases: no stabilization, mechanical stabilization, and the combination of mechanical stabilization and digital stabilization, which are depicted in Figure 7. The periodic motion lasted for 0.67 seconds at a frequency of 1.5 Hz. The symbol of the cross shape was 0.015 m in front of the camera. The resolution of the camera was 1920 × 1080 pixels. To avoid shadows after motion compensation, the image was cropped from the center to 800 × 600 pixels. The middle picture shows the initial position, the left and right sides were moving to the left and right of this, respectively.

Obviously, tilting, offset, and ghosting phenomenon are very pronounced when there is no visual sensor stabilization system. The center point of the cross has vanished from the frame in the worst case. The 1-DOF mechanical stabilization system, by contrast, has a noticeable stabilizing effect. The center of the cross-shaped mark always shows clearly in the graphic. In addition, the degree of tilting is also greatly improved in the visual sensor stabilization platform that combines mechanical stabilization with digital stabilization. In general, the target detection bounding box is a quadrangle. Thus, digital stabilization can weaken the influence of the roll disturbance, it reflects the real shape under view, and enhances the recognition accuracy.

### 5.3. Target Recognition

**Figure 8:**
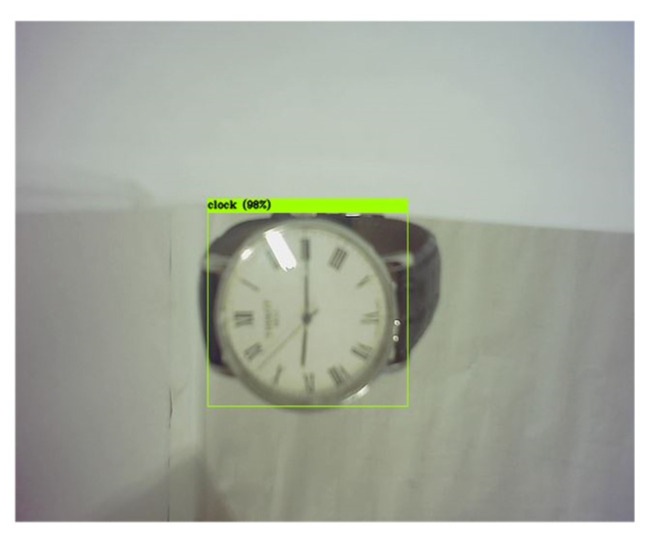
An example of the target recognition experiment, in which the quadrangle is the recognition frame and 98% is the confidence.

**Figure 9:**
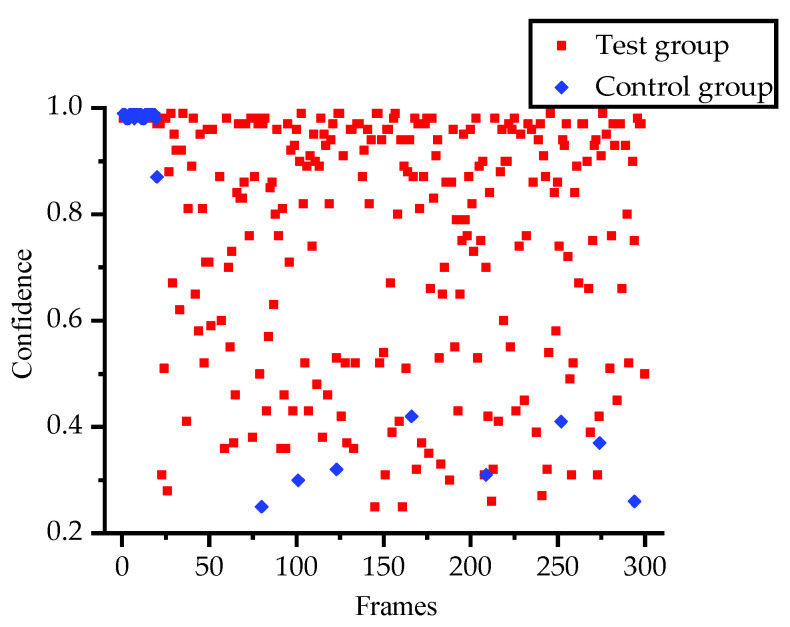
Confidence contrast scatter diagram, where the control group is the system without any stabilization methods, the test group is the visual sensor stabilization system, and the first period is stationary.

With regard to the ultimate goal of the visual sensor stabilization of the platform, the effectiveness of the visual sensor stabilization system in target recognition during a part of the process was verified. First, the system without any stabilization methods was used as a control group, while the stabilization system constructed in this paper served as a test group. Secondly, the YOLOv3-tiny algorithm was used based on the frame rate of the current camera, which was 30 fps although this might be higher in the future [39]. It worked with excellent speed, and was able to meet the requirements. The identification object was the clock, which was in the COCO database [40]. Finally, the experimental layout is illustrated in Figure 8, in conjunction with the confidence contrast scatter diagram as shown in Figure 9. The recognition process only exhibited objects detected with a confidence of 0.25 or higher, and the processing time was 10 s.

As seen in Figure 9, the number of frames recognized by the clock after stabilization was greater than that of the system without stabilization. The reason is that the image blurring caused by high-frequency motion reduces the accuracy of the algorithm, which means the control group is unable to recognize the object in motion. Therefore, choosing a better visual sensor and a more suitable algorithm can significantly improve the effect. To summarize, the visual sensor stabilization platform described in this work significantly improved the accuracy of target recognition under the condition of oscillatory motion.

## 6. Conclusions and Future Work

This paper proposed a novel 1-DOF visual sensor stabilization platform for obtaining continuous steady image sequences in real-time for the 3-D motion of the shark-like robotic fish. An improved LADRC that combines DCI and LADRC was employed because it strikes a balance between the control error and dynamic performance in practice. It takes into account the characteristics of the disturbance and improves the performance of the LADRC. The MAE and RMSE of the improved LADRC were greatly enhanced, and were 5.6715° and 4.8038° respectively. Furthermore, a digital stabilization algorithm that takes advantage of IMUs was applied, which improves the effect of the roll direction stabilization. The tilting phenomena was greatly improved. Ultimately, the effectiveness of the visual sensor stabilization platform was verified through different experiments. In addition to the first stationary cycle, the remaining fourteen motion cycles increased the detection rate from 2.86% to 87.86%. The obtained results offer a valuable insight into the construction of underwater visual sensing systems, and shed light on the updated design and control of oscillatory structures for better mobility and target tracking performance.

Ongoing and future work will focus on enhancing the algorithm stability and response speed. With regard to digital stabilization, the IMUs data can be combined with ranging sensors, such as ultrasonic sensors to compensate for the offset due to the disturbance. Furthermore, in the visual sensor stabilization systems of other types of robots, the components of mechanical stabilization could be optimized in dolphin-like swimming robots with larger interior space.

## Figures and Tables

**Table 1:** Description of the formulaic terms.

Term	Description
$v_{1}$, $v_{2}$	Target Euler angle and angular velocity
*y*	Euler angle measured by IMUs
*h*	Sampling period
$\beta_{01}$, $\beta_{02}$, $\beta_{03}$	Observer gains
*b*	Input gain
$z_{1}$, $z_{2}$	Observer angle and angular velocity
$z_{3}$	Total disturbance
${\tilde{e}}_{1}$	Tracking deviation
$w_{0}$	Bandwidth of the observer

**Table 2:** Stabilization precision of visual sensor platform with different methods.

Method	APR (°)	Range (°)	MAE (°)	RMSE (°)
PD	20.0357	23.2881	7.0878	7.5673
LADRC	14.7707	38.5620	7.0657	9.5887
Switching system	16.7520	36.2005	6.8003	8.7368
Improved LADRC	15.6724	23.4746	5.6715	4.8038
